# Combined De-Repression of Chemoresistance Associated Mitogen-Activated Protein Kinase 14 and Activating Transcription Factor 2 by Loss of microRNA-622 in Hepatocellular Carcinoma

**DOI:** 10.3390/cancers13051183

**Published:** 2021-03-09

**Authors:** Valerie Fritz, Lara Malek, Anne Gaza, Laura Wormser, Majken Appel, Andreas E. Kremer, Wolfgang E. Thasler, Jürgen Siebler, Markus F. Neurath, Claus Hellerbrand, Anja K. Bosserhoff, Peter Dietrich

**Affiliations:** 1 Institute of Biochemistry, Emil-Fischer-Zentrum, Friedrich-Alexander-University Erlangen-Nürnberg, 91054 Erlangen, Germany; valerie.fritz@fau.de (V.F.); lara.malek@web.de (L.M.); anne.gaza@uk-erlangen.de (A.G.); laura.wormser@fau.de (L.W.); majken.appel@fau.de (M.A.); claus.hellerbrand@fau.de (C.H.); anja.bosserhoff@fau.de (A.K.B.); 2 Department of Medicine 1, University Hospital Erlangen, Friedrich-Alexander-University Erlangen-Nürnberg, 91054 Erlangen, Germany; andreas.kremer@uk-erlangen.de (A.E.K.); juergen.siebler@uk-erlangen.de (J.S.); Markus.Neurath@uk-erlangen.de (M.F.N.); 3 Deutsches Zentrum für Immuntherapie (DZI), Friedrich-Alexander-University Erlangen-Nürnberg and University Hospital Erlangen, 91054 Erlangen, Germany; 4 Department of General and Visceral Surgery, Red Cross Hospital of Munich, 80634 Munich, Germany; wolfgang.thasler@swmbrk.de; 5 Comprehensive Cancer Center (CCC) Erlangen-EMN, 91054 Erlangen, Germany

**Keywords:** liver cancer, HCC, Hepatocellular carcinoma, microRNA, miR-622, RAS, MAPK14

## Abstract

**Simple Summary:**

Novel approaches to target RAS-signaling have led to the so called “RAS renaissance”. In HCC, it was demonstrated that wild-type RAS-RAF-ERK-signaling strongly contributes to HCC progression and drug resistance. In this context, microRNA-622 has emerged as one of the most promising tumorsuppressive microRNAs, targeting RAS-signaling in HCC. However, the majority of microRNA-622 target genes remained elusive. Moreover, limited chemoresponse of HCC was demonstrated by activation of MAPK14-ATF2-signaling which represents a RAS-collateral-pathway, but the expression and regulation of both MAPK14 and ATF2 remained to be clarified. In this study, we demonstrated co-overexpression of MAPK14 and ATF2 in HCC in vitro and in vivo. Moreover, in contrast to “one-microRNA-one-target” interactions, we identified common binding sites for microRNA-622 in both synergistic drug resistance-associated genes–MAPK14 and ATF2. Our data revealed that microRNA-622 functions as a superior pathway regulator inducing de-repression of the MAPK14-ATF2-axis in HCC.

**Abstract:**

Chemoresistance is a major hallmark driving the progression and poor prognosis of hepatocellular carcinoma (HCC). Limited chemoresponse of HCC was demonstrated to be mediated by mitogen-activated protein kinase 14 (MAPK14) and activating transcription factor 2 (ATF2). Recently, we have demonstrated loss of control of RAS-RAF-ERK-signaling as a consequence of miR-622 downregulation in HCC. However, the majority of target genes of this potent tumorsuppressive microRNA had remained elusive. The MAPK14-ATF2-axis represents a collateral pathway ensuring persisting ERK-activation in the presence of sorafenib-mediated RAF-inhibition. In contrast to the function of the MAPK14-ATF2-axis, both the expression and regulation of MAPK14 and ATF2 in human HCC remained to be clarified. We found combined overexpression of MAPK14 and ATF2 in human HCC cells, tissues and in sorafenib resistant cell lines. High expression of MAPK14 and ATF2 was associated with reduced overall survival in HCC patients. Deciphering the molecular mechanism promoting combined upregulation of MAPK14 and ATF2 in HCC, we revealed that miR-622 directly targets both genes, resulting in combined de-repression of the MAPK14-ATF2-axis. Together, miR-622 represents a superior regulator of both RAS-RAF-ERK as well as MAPK14-ATF2-signaling pathways in liver cancer.

## 1. Introduction

Hepatocellular carcinoma (HCC) is considered as one of the deadliest cancer types worldwide [1,2,3]. Chemoresistance represents a major hallmark driving the progression and poor prognosis of HCC [4,5].

Limited response of liver cancer cells to first-line systemic therapeutic options such as receptor-tyrosine-kinase inhibitors (i.e., lenvatinib and sorafenib) was demonstrated by our group to be mediated by RAS-activated collateral pathways ensuring persisting ERK-activation [6,7,8,9,10]. Recently, in vivo RNAi screening had identified activation of the mitogen-activated protein kinase 14 (MAPK14)-activating transcription factor 2 (ATF2)-axis as a novel mechanism driving sorafenib resistance in HCC [11]. Upon phosphorylation by MAPK14, ATF2 can function as a tumor promotor [11,12]. Moreover, MAPK14 ensures persisting ERK-activation in the presence of sorafenib-mediated RAF-inhibition [11]. However, apart from these functional analyses, the expression and regulation of the MAPK14-ATF2-axis remained unclear in HCC.

In this study, we aimed at deciphering the expression, cellular localization and molecular regulation of MAPK14 and ATF2 in HCC.

## 2. Results

### 2.1. Combined Overexpression of MAPK14 and ATF2 in HCC Cells and in Sorafenib Resistance

**Figure 1:**
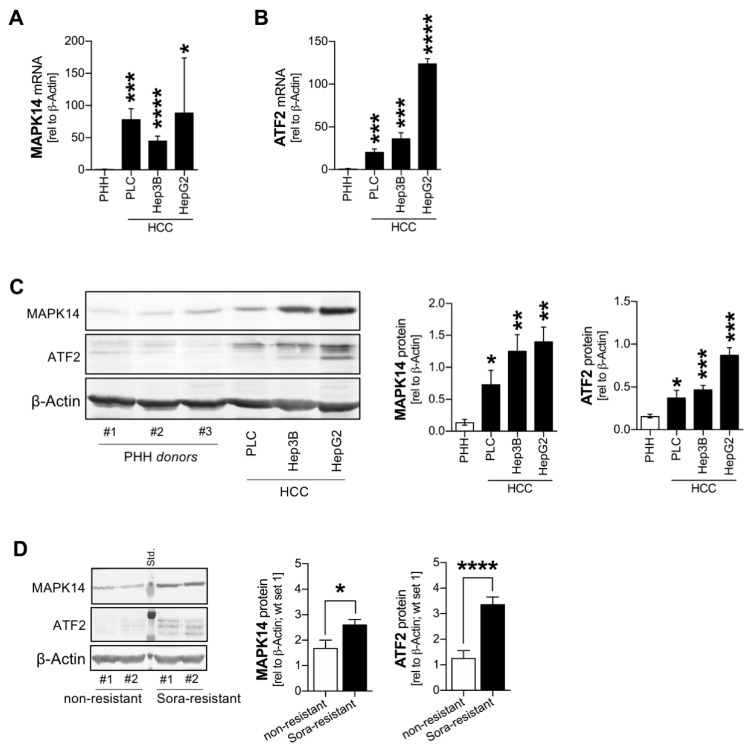
Combined overexpression of MAPK14 and ATF2 in HCC cells and in sorafenib resistance. (**A**) Quantitative RT-PCR analysis of MAPK14 mRNA levels in primary human hepatocytes (PHH) (*n* = 4) as compared with human HCC cell lines (PLC (*n* = 3), Hep3B (*n* = 3), HepG2 (*n* = 3)). (**B**) Quantitative RT-PCR analysis of ATF2 mRNA levels in primary human hepatocytes (PHH) (*n* = 4) as compared with human HCC cell lines (PLC (*n* = 3), Hep3B (*n* = 3), HepG2 (*n* = 3)). (**C**) Representative image (including three different donors of PHH (d#1, d#2, d#3) and (densitometric) Western blot quantification displaying MAPK14 and ATF2 protein levels in PHH (*n* = 4) as compared with human HCC cell lines (PLC (*n* = 3), Hep3B (*n* = 3), HepG2 (*n* = 3)). (**D**) Representative image and (densitometric) Western Blot analysis of MAPK14 and ATF2 protein levels in non-resistant (*n =* 3) as compared with Sorafenib-resistant (Sora-resistant) Hep3B cells (*n = 3*). Data are presented as mean ± SEM. Statistical significance was determined by 2-tailed, unpaired *t*-test (**A**–**D**). * *p* < 0.05, ** *p* < 0.01, *** *p* < 0.001, **** *p* < 0.0001.

First, the expression of MAPK14 and ATF2 was analyzed in human HCC in vitro. Primary human hepatocytes were compared with human HCC cell lines (PLC, Hep3B, HepG2). Quantitative RT-PCR analyses revealed that both MAPK14 (Figure 1A) and ATF2 (Figure 1B) were strongly increased in HCC cells. Combined upregulation of MAPK14 and ATF2 in liver cancer cells was also determined on the protein level applying Western blot analysis (Figure 1C). The MAPK14-ATF2-axis was recently shown to promote sorafenib resistance [11]. Accordingly, as compared with non-resistant HCC cells, established sorafenib-resistant cell lines [6,7,8] showed even stronger expression of both MAPK14 and ATF2 (Figure 1D).

Together, the resistance associated MAPK14-ATF2-axis was overexpressed in human HCC cells and in sorafenib resistance in vitro.

### 2.2. Combined Upregulation of MAPK14 and ATF2 in HCC In Vivo

**Figure 2:**
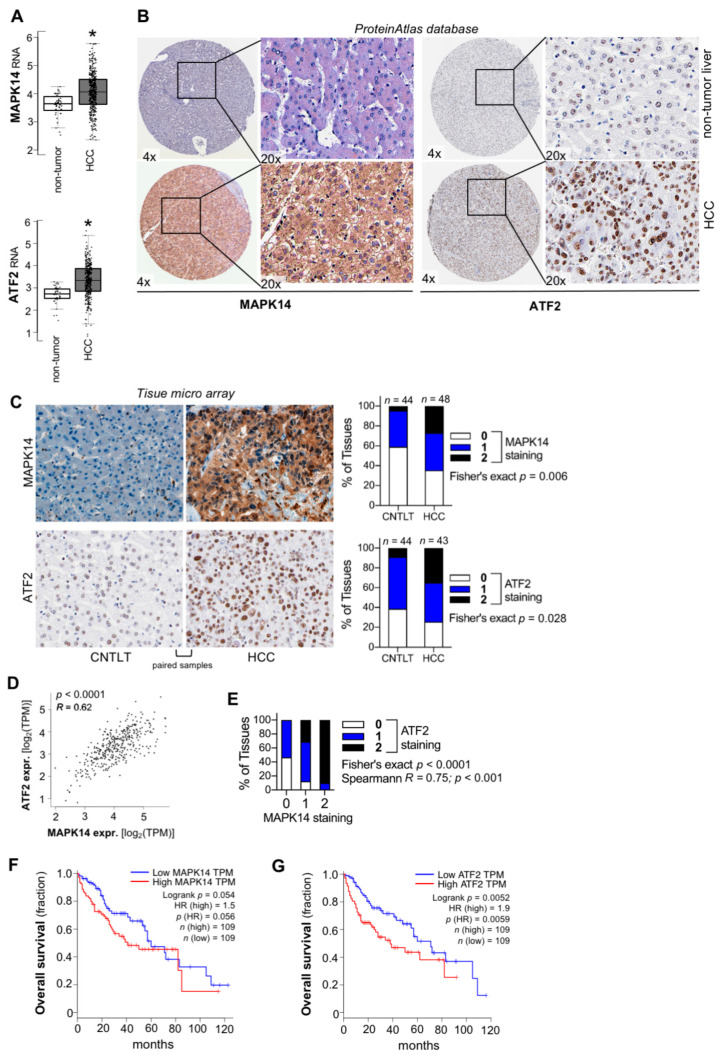
Combined upregulation of MAPK14 and ATF2 in HCC in vivo. (**A**) Transcriptomics data of MAPK14 (upper panel) and ATF2 (lower panel) RNA expression in HCC (*n* = 369) and non-tumorous liver tissues (*n* = 50) derived from TCGA-data applying the Gene Expression Profiling Interactive Analysis (GEPIA) database. (**B**) Representative immunohistochemistry images depicting MAPK14 (left side) and ATF2 (right side) protein expression and cellular localization patterns in HCC as well as low expression in non-tumorous liver tissues applying the ProteinAtlas database. (**C**) Representative immunohistochemical images (left side) and quantification (right side) of MAPK14 and ATF2 protein levels applying tissue micro array (TMA) analysis containing HCC and corresponding non-tumorous liver tissue (CNTLT) derived from human donors. Staining intensity and percentage of positive cells were incorporated into a semi-quantitative score describing “0” (very low/no expression), “1” (low/moderate expression) and “2” (strong expression). (**D**) Correlated MAPK14 and ATF2 RNA expression levels (log_2_(TPM)) in HCC tissues. “The Cancer Genome Atlas” (TCGA) derived data were used applying the Gene Expression Profiling Interactive Analysis (GEPIA) database. (**E**) Immunohistochemical analysis and correlation of MAPK14 and ATF2 expression levels applying tissue micro arrays (staining intensity and percentage of positive cells for both MAPK14 and ATF2 were incorporated into a semi-quantitative score describing “0” (very low/no expression), “1” (low/moderate expression) and “2” (strong expression)) (*n* = 43). (**F**,**G**) TCGA-derived datasets and the Gene Expression Profiling Interactive Analysis (GEPIA) database were used for overall survival analysis comparing high and low MAPK14 (F) and ATF2 (G) levels, respectively, in HCC patients. “HR”: Hazard ratio. Data are presented as mean ± SEM. Statistical significance was determined by 2-tailed, unpaired *t*-test (**A**), two-sided Fisher’s exact test together and/or Spearman correlation analysis (**C**,**E**), and Pearson correlation (**D**). Survival analysis was performed computationally applying log-rank testing and hazard ratio estimates (**F**,**G**). * *p* < 0.05.

Next, expression levels of the MAPK14-ATF2-axis were determined in vivo. Analyses of transcriptomics data derived from “The Cancer Genome Atlas” (TCGA) confirmed upregulation of both MAPK14 and ATF2 RNA levels in HCC tissues (Figure 2A). Applying the human ProteinAtlas database [13,14,15], cytoplasmatic staining of MAPK14 and a predominant nuclear staining pattern of ATF2 were detected in HCC tissues (Figure 2B). The typical cellular localization patterns of MAPK14 and ATF2 were also confirmed in non-HCC cancer cells (Figure S1A,B). Moreover, marked overexpression of both MAPK14 and ATF2 protein levels was found in HCC as compared with non-tumorous liver tissues (Figure 2B).

To extend these findings, expression levels of MAPK14 and ATF2 were determined applying a tissue micro array (TMA) containing HCC tissues and corresponding non-tumorous liver tissues of HCC-patients [6,16,17]. Here, combined upregulation of both MAPK14 and ATF2 protein levels were confirmed in HCC tissues (Figure 2C; Table S1). Furthermore, analysis of TCGA-derived data revealed a strong and significant correlation between MAPK14 and ATF2 expression levels in human HCC, further pointing to a common regulatory mechanism of both genes (Figure 2D). Co-expression of MAPK14 and ATF2 in HCC tissue samples was also confirmed on the protein level applying tissue micro arrays (Figure 2E; Tables S2 and S3).

Moreover, high expression levels of MAPK14 or ATF2 were associated with advanced tumor stages (Figure S1C,D) and a more de-differentiated histological grading (Tables S2 and S3). Furthermore, high as compared with low MAPK14 expression levels were slightly but non-significantly associated with reduced overall patient survival (Figure 2F; Figure S1E). In contrast, high ATF2 levels were significantly and strongly associated with reduced survival in HCC patients (Figure 2G; Figure S1F).

In summary, next to the in vitro findings, combined overexpression of the MAPK14-ATF2-axis in HCC was confirmed in patient-derived tissue samples. Together, these data suggested a combined regulatory mechanism for MAPK14 and ATF2 overexpression in liver cancer.

### 2.3. Loss of microRNA-622 Mediates De-Repression of the MAPK14-ATF2-Axis in HCC

**Figure 3:**
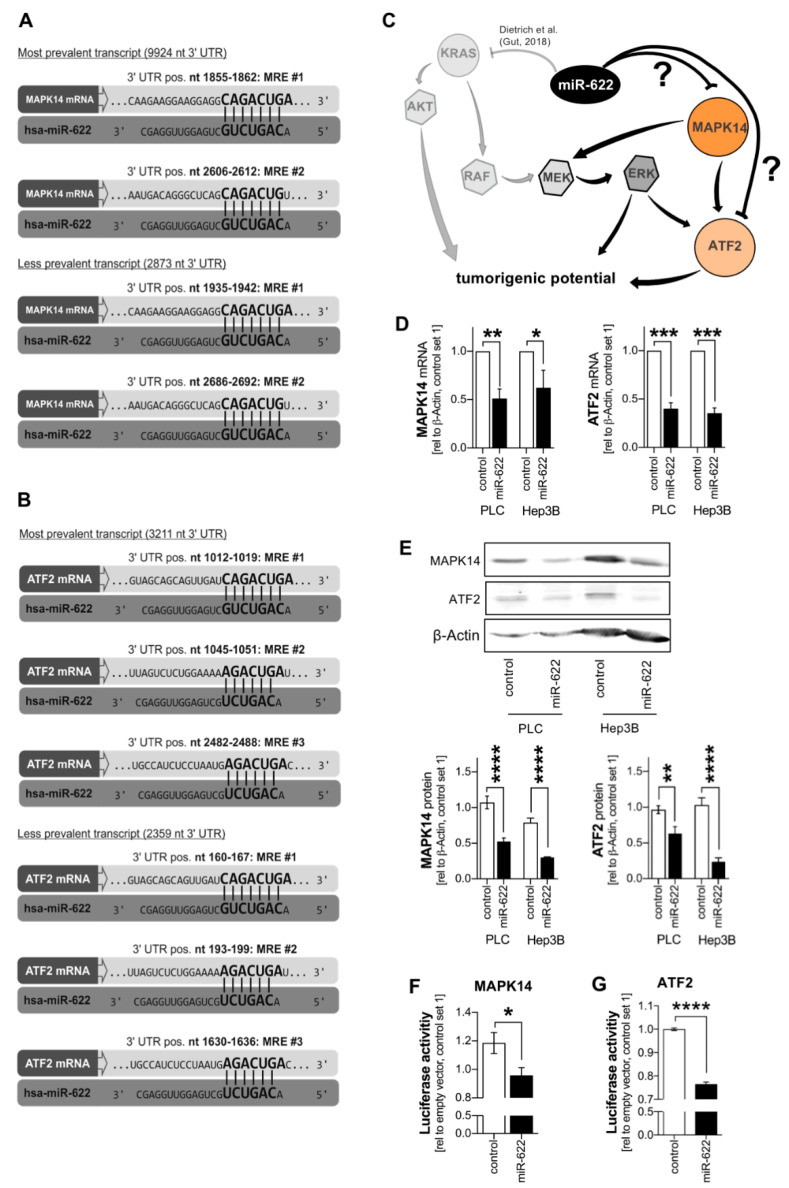
Loss of microRNA-622 mediates de-repression of the MAPK14-ATF2-axis in hepatocellular carcinoma. (**A**,**B**) Several microRNA recognition elements (MRE) representing potential miR-622-binding sites were identified in MAPK14 and ATF2 3′UTRs applying the “TargetScan 7.2” database. (**C**) Hypothesis of potential impact of miR-622 on both MAPK14-ATF2-signaling and RAS-RAF-ERK-signaling in the sense of a superior regulator of resistance-associated pathways. The MAPK14-ATF2 signaling pathway can bypass the RAS-RAF-pathway thereby inducing drug resistance [11]. KRAS had been identified by our group to be regulated by miR-622 [6]. (**D**) Quantitative RT-PCR analysis of MAPK14 and ATF2 mRNA levels in control-transfected as compared to miR-622-transfected different HCC cell lines (PLC (*n =* 4)*;* Hep3B (*n =* 4)). (**E**) Representative Western blot images and (densitometric) analysis of MAPK14 and ATF2 protein levels in control-transfected as compared to miR-622-transfected different HCC cell lines (PLC (*n =* 4)*;* Hep3B (*n =* 4)). (**F**) Luciferase MAPK14 3′UTR-reporter (containing a conserved miR-622 MRE) activity in control-miR (CTR) as compared with miR-622-mimic (622)-transfected HCC cells (PLC) (*n* = 4). (**G**) Luciferase ATF2 3′UTR-reporter (containing the two conserved miR-622 MREs (MRE#1 and MRE#2)) activity in control-miR (CTR) as compared with miR-622-mimic (622)-transfected HCC cells (PLC) (*n* = 3). Data are presented as the mean ± SEM. Statistical significance was determined by 2-tailed, unpaired *t*-test (**D**–**G**). * *p* < 0.05, ** *p* < 0.01, *** *p* < 0.001, **** *p* < 0.0001.

Combined upregulation of both MAPK14 and ATF2 in HCC on both the RNA and the protein level pointed towards transcriptional or microRNA-induced regulation mediated by a common transcription factor or a common microRNA, respectively. Applying microRNA-target interaction databases (“miRTarBase 2020”; “miRDB”; “TargetScan”; “miRNAMAP 2.0”) [18,19,20,21], we identified several conserved binding sites for one microRNA–miR-622–in the 3′untranslated regions (UTR) of both MAPK14 (Figure 3A) and ATF2 (Figure 3B) transcript variants. Of note, loss of KRAS control and subsequent RAF-ERK-activation as a consequence of the downregulated, strongly tumorsuppressive miR-622 was recently demonstrated by our group to induce sorafenib resistance in HCC [6,10,22]. Since MAPK14-mediated activation of MEK-ERK-signaling can functionally overcome sorafenib resistance [11], we hypothesized that miR-622 might represent a superior regulator of both RAS-RAF-ERK as well as MAPK14-ATF2-signaling pathways in liver cancer. (Figure 3C).

Quantitative RT-PCR analyses applying two different HCC cell lines (PLC, Hep3B) demonstrated that re-expression of miR-622 significantly reduced mRNA expression levels of MAPK14 and ATF2 (Figure 3D). Additionally, on the protein level, miR-622 re-expression was sufficient to strongly and simultaneously reduce MAPK14 and ATF2 expression (Figure 3E).

Mechanistically, a direct miR-622-MAPK14-interaction has been completely elusive. To mechanistically validate the novel miR-622-MAPK14 interaction, a luciferase reporter gene construct containing one of the conserved miR-622 microRNA recognition elements (MRE#1) of MAPK14 was generated. Co-transfection of the luciferase reporter comprising the MAPK14-MRE#1 sequence together with miR-622 re-expression significantly reduced luciferase activity in HCC cells as compared with the respective control (Figure 3F). Moreover, ATF2 had recently been confirmed to directly interact with miR-622 (applying luciferase reporter activity assays) in non-HCC (i.e., glioma) cancer cells [23]. In HCC cells (PLC), applying a luciferase reporter gene construct comprising the two predicted miR-622 response elements MRE#1 and MRE#2 of ATF2 was generated, and subsequent luciferase activity assays revealed a direct miR-622-ATF2-interaction (Figure 3G).

In summary, both MAPK14 and ATF2 were identified as novel target genes of miR-622 in HCC, outlining this microRNA as a master regulator of the drug resistance associated MAPK14-ATF2 and RAS-RAF-ERK pathways.

## 3. Discussion

It has been demonstrated by our group and others that microRNAs (miRs) represent powerful small RNA molecules that modulate drug resistance related features in HCC [10,24]. In recent years, miR-622 has emerged as one of the most promising tumorsuppressive microRNAs, which is underscored by multiple studies addressing its potent role in diverse types of cancer [6,10,22,25,26,27,28,29,30,31,32].

However, in contrast to other cancer types, so far only three studies have identified and experimentally proved target genes of miR-622 in liver cancer (i.e., mitogen-activated protein 4 kinase 4 (MAP4K4), CXC chemokine receptor 4 (CXCR4), and kirsten rat sarcoma (KRAS) [6,32,33]. Regarding the possibility that one single microRNA can regulate a target gene network composing of hundreds of mRNAs [10,34], the vast majority of miR-622-targets in liver cancer (and also other cancer types) remained elusive.

Applying comprehensive in silico prediction-database screening, we identified common miR-622 binding sites in both MAPK14 and ATF2 3′UTRs. To the best of our knowledge, a common regulation of directly cooperating genes by one microRNA has not been described before in HCC.

Subsequently, MAPK14 and ATF2 were both identified as novel, direct target genes of miR-622 in liver cancer. Applying quantitative RT-PCR analysis, Western blot analysis and luciferase reporter gene assays, we demonstrated that miR-622-overexpression was sufficient to simultaneously depress both MAPK14 and ATF2, thereby representing a promising and potentially therapeutic approach to tackle this resistance-associated signaling-axis from two sides. Accordingly, proliferation analysis revealed that simultaneous re-expression of miR-622 markedly restored sorafenib-efficacy in sorafenib-resistant HCC cells (Figure S2A,B).

The potential mechanistic advantage of the observed, simultaneous upregulation of MAPK14 and ATF2 mediated by loss of miR-622 in sorafenib-resistant HCCs is of special interest. In this regard, co-overexpression of cooperating partner genes was described in diverse cancer types and represents a well-known hallmark contributing to synergistic pathway activation which can give a competitive edge for cancer cells in specific circumstances including drug resistance. For example, co-overexpression of Janus kinase 2 and signal transducer and activator of transcription 5a was shown to affect mammary cancer cells via the epithelial–mesenchymal transition-pathway [35]. Likewise, co-overexpression of HER2/HER3 is a predictor of impaired survival in breast cancer patients [36]. Moreover, co-overexpression of fibroblast growth factor receptors 1, 2 and 4 revealed prognostic significance in gastric cancer [37]. Furthermore, co-overexpression of YAP and TAZ was shown to be an independent predictor of prognosis for patients with colorectal cancer, and may account for the higher proliferation, metastasis, and poor survival outcome of these patients [38]. Additionally, in renal cell carcinoma, concomitant overexpression of the EGFR and erbB-2 was correlated with dedifferentiation and metastasis [39]. Together, numerous studies support that cancer cells can benefit from simultaneous co-overexpression of cooperating pathway genes.

The MAPK14-ATF2-axis was functionally shown to represent a collateral pathway ensuring persisting ERK-activation in the presence of sorafenib-mediated RAF-inhibition, thereby driving drug resistance [11]. Therefore, combined de-repression of both MAPK14 and ATF2 as mediated by loss of miR-622 could mechanistically contribute to stronger activation of the beneficial MAPK14-ATF2-axis as compared with alternative pathways of ATF2-activation: ATF2 consists of multiple domains; the most prominent are the N-terminally located transactivation domain, the zinc finger, the bZIP domain and the nuclear localization and nuclear export signals [40]. Likewise, ATF2 can be phosphorylated by numerous kinases including MAPK14, ERK, PKC, JNK and also by ATM at different sites. Depending on the upstream activation pathway, ATF2 is then translocated into the nucleus where it either forms a homodimer or heterodimer, either with intra-family proteins (ATF3, CRE-BPa or JDP2) or other bZIP proteins [40]. Dependent on its dimerization partner, ATF2 can bind to cAMP-response elements or to the AP-1 binding motif together with c-JUN [40]. Off note, different upstream activation pathways of ATF2 induce different, partly divergent downstream effects, and therefore, ATF2 is considered as a transcription factor that elicits both oncogenic or tumor suppressor activities [40,41]. Interestingly, while mitogen-activated protein kinases (JNK, ERK and MAPK14) induced oncogenic activation of ATF2, ATM-mediated ATF2-phosphorylation was shown to prevent carcinogenesis [40]. Therefore, ATM, a stress-associated protein kinase which is also activated by, e.g., DNA double-strand breaks, acts as a tumorsuppressive gate keeper also via activation of ATF2 [40]. Like ATM, MAPK14 also represents a kinase which is strongly activated by stress signals and, e.g., inflammatory cytokines, but however activates oncogenic downstream-signaling [42]. In conclusion, in the presence of RAF-ERK-inhibition by sorafenib (which is also known as a strong activator of diverse cell-stress-associated pathways), the oncogenic MAPK14-ATF2-pathway might compete with tumorsuppressive ATM-ATF2-signaling. In this scenario, co-upregulation of MAPK14 (together with ATF2) might enable the competitive edge for MAPK14- as compared to ATM-mediated ATF2 activation, thereby driving tumor progression and drug resistance [40]. Together, combined de-regulation of MAPK14 and ATF2 by loss of miR-622 might be specifically beneficial for cancer cells under stress-inducing conditions including chemotherapy. According to this hypothesis, combined overexpression of the resistance associated MAPK14-ATF2-axis was shown in HCC cells and tissues and also in sorafenib resistant HCC cells in our study.

First-generation inhibitors of MAPK14 have already been tested in clinical trials. However, these inhibitors lacked specificity for MAPK14 and revealed several toxicities [43]. In contrast, second-generation inhibitors of MAPK14 showed high target specificity and a very low toxicity profile [44,45]. Meanwhile, some of these inhibitors have already completed phase I and II trials for non-cancer related diseases (e.g., NCT00729209, NCT01366014, NCT00383188 and NCT00559910). Likewise, activated MAPK14 is considered as a druggable target in cancer including pancreatic adenocarcinoma [46]. Therefore, several novel specific inhibitors of MAPK14 are currently tested in phase I and II trials for diverse non-HCC cancer types like metastatic breast cancer and adult glioblastoma [47]. Our findings underscore that MAPK14 inhibitors might represent a promising option in HCC and should therefore be evaluated in clinical trials. Moreover, ATF2 is considered as a major driver of cancer progression [40] and might serve as a promising future therapeutic target in HCC and other types of cancer. In conclusion, combinations of specific MAPK14- and ATF2-inhibitors might outline a synergistic therapeutic approach in (liver) cancer.

Interestingly, we found that high as compared with low MAPK14 levels were only slightly but not significantly associated with reduced overall survival, while high ATF2 expression was confirmed in two datasets to be significantly associated with reduced survival. This discrepancy might be potentially be explained by the MAPK14-ATF2-signaling-axis, since MAPK14 acts upstream of ATF2, while ATF2 phosphorylation and subsequent activation can not only be induced by MAPK14, but also by, e.g., activated ERK. Since the ERK-pathway can alternatively induce ATF2 activation, convergence of both RAS-ERK-signaling and MAPK14-signaling into ATF2-activation might explain stronger effects on patient survival mediated by ATF2 as compared with its partner MAPK14. However, the indispensable importance of MAPK14 to activate ATF2 is brought into action when the RAS-ERK-signaling is blocked, e.g., by tyrosine kinase inhibitors like sorafenib/lenvatinib. In conclusion, futures large patient cohort studies containing exclusively sorafenib/lenvatinib-resistant or -treated patients should be analyzed to reveal whether in this situation, also high MAPK14 is significantly associated with reduced survival.

Next to the MAPK14-ATF2-axis, KRAS-signaling had been shown to be regulated by miR-622 and to be associated with sorafenib resistance by our group [6,11]. Together, our current study outlines this microRNA as a master regulator of the drug resistance associated RAS-RAF-ERK and MAPK14-ATF2 pathways.

## 4. Material and Methods

### 4.1. Human Cells and Cell Culture

The human hepatocellular carcinoma (HCC) cell lines used in this study (PLC (ATCC CRL-8024), Hep3B (ATCC HB-8064) and HepG2 (ATCC HB-8065)) were described elsewhere [6,17]. Sorafenib-resistant HCC cells were generated by incubation with stepwise increasing doses of sorafenib (0.5–1.0 µM per week) up to 8.0 µM. Sorafenib (“Nexavar”) was purchased from Cayman Chemical (Ann Arbor, MI, USA). Real-time cell proliferation analysis was performed applying the xCELLigence system (Roche, Mannheim, Germany) as described previously [30]. Primary human hepatocytes (PHH) were isolated and cultured as described by Lee et al. [48].

### 4.2. Human Liver Tissue

Human HCC tissues and non-tumorous liver tissues (CNTLT) specimens were obtained from patients undergoing surgical procedures. The tissue samples were instantly snap-frozen and stored at −80 °C. Moreover, paraffin-embedded tissues were used to construct tissue micro arrays (TMA) for immunohistochemical analysis as described [6,17,49]. Immunohistochemical analysis of MAPK14 and ATF2 expression levels applying tissue micro arrays (staining intensity and percentage of positive cells for both MAPK14 and ATF2) were incorporated into a semi-quantitative score describing “0” (very low/no expression), “1” (low/moderate expression) and “2” (strong expression). The informed patients’ consent were collected and obtained by the Biobank at the Hospital of the Ludwig-Maximilians-University Munich, which is subject to the guidelines of the non-profit state-controlled Human Tissue and Cell Research (HTCR) foundation [49].

### 4.3. RNA Isolation and Expression Analysis

RNA isolation and reverse transcription were performed as described elsewhere [50]. Quantitative reverse-transcription polymerase chain reaction (qRT-PCR) was performed using specific primers on a Lightcycler 480 system (Roche, Mannheim, Germany). The following primers were used: β-Actin (5′-CTA CGT CGC CCT GGA CTT CGA GC-3′ and 5′-GAT GGA GCC GCC GAT CCA CAC GG-3′), 18S (5′-TCT GTG ATG CCC TTA GAT GT CC-3′ and 5′-CCA TCC AAT CGG TAG TAG CG-3′), MAPK14 (5′-GGT AAA ATC TCG GCT CTC GG-3′ and 5′-CTC CGG CGC TCA AGA CTG-3′) and ATF2 (5′-GGC ATT CAA GCA GGA TTC CAC-3′ and 5′-TTG GAA CTT AGG CAG GAG GG-3′).

### 4.4. Transfection of HCC Cells with microRNA-622

2–3 × 10^5^ cells were seeded per well of a six-well culture plate. MicroRNA (miRNA) mimics (Ambion by ThermoFisher Scientific, Waltham, MA, USA) were described before [8,30]. Extraction of total RNA and protein followed 48 to 72 h after transfection.

### 4.5. Cloning, Luciferase Reporter Gene Assays & RNA Pulldown

The wild-type sequence of the MAPK14 3′untranslated region (UTR) spanning the required miR-622 response element (MRE#1) was amplified applying PCR and HCC cell-derived cDNA using the Phusion High-Fidelity DNA Polymerase kit (ThermoFisher Scientific, Waltham, MA, USA) and the following primers: 5′-CAG TCT AGA CCC TGA GTC AAC TGG AGC AA-3′ and 5′-CAG TCT AGA TGA AAG GGG GAG AGT CC CG-3′). For ATF2, the wildtype ATF2 3′ UTR sequence comprising the two predicted miR-622 response elements MRE#1 and MRE#2 was amplified from HCC cell-derived template cDNA by using the following primers: 5′-CAG TCT AGA CCT GCA GTA CAA CAG TTT TA GA-3′ and 5′-CAG TCT AGA CTA CTA CCG TCA CAG TTG AG GG-3′.

The generated amplicons were inserted into the Luciferase 3′ UTR of a pGL3-Promoter Firefly Luciferase reporter vector (Promega Corporation, Madison, WI, USA). All plasmid sequences were verified by sequencing. The obtained Firefly Luciferase reporter constructs (FLuc) were co-transfected with pre-miR-622 or non-targeting control-microRNA (Ambion by ThermoFisher Scientific, Waltham, MA, USA) and the wild-type Renilla reporter pRL-TK (RLuc; Promega Corporation, Madison, WI, USA) for normalization. Lipofectamine 2000 Transfection Reagent (ThermoFisher Scientific, Waltham, MA, USA) was used for transfection of cells. FLuc signals were normalized to the corresponding RLuc signals by calculating the RLuc/FLuc ratio and correcting for differences in transfection efficiencies. Luciferase assays were performed as described [6].

### 4.6. Western Blotting

Cell lysis, protein isolation and Western blots were carried out as specified in other studies [7,8,16]. The following primary antibodies were used: Anti-β-actin (1:5000 dilution) (Sigma-Aldrich, St. Louis, MO, USA), anti-MAPK14 in (1:1000 dilution) (Cell Signaling, Danvers, MA, USA), anti-ATF2 in (1:1000 dilution) (Cell Signaling, Danvers, MA, USA). β-actin served as a reference for normalization. Immunoreactions were visualized applying the BCIP/NBT kit (Invitrogen by Thermo Fisher Scientific, Waltham, MA, USA). Densitometric analysis of scanned Western blot images was performed using “ImageJ” (National Institutes of Health, Bethesda, MD, USA).

### 4.7. Immunohistochemistry and Immunofluorescence Analysis

Immunohistochemistry analysis was performed applying tissue micro arrays comprising human patient derived liver cancer tissue samples [7,8,16]. Immunohistochemical staining was analyzed semi-quantitatively and the according scores were established for each antibody as previously described [6] and in the respective figure legends.

Immunofluorescence assays were performed as described [51]. Briefly, after seeding the cells in chamber slides (20,000 cells per chamber) and 24 h incubation, cells were fixed with methanol-acetic acid and afterwards permeabilized using 0.1% Triton-X-100. Subsequently, the cells were blocked for 1 h using 1% bovine serum albumin/PBS, and subsequently incubated over night with specific primary antibodies. The following primary antibodies were used for immunohistochemistry/immunofluorescence: anti-MAPK14 (1 in 50 dilution) (Cell Signaling, Danvers, MA, USA); anti-ATF2 (1:100 dilution) (Cell Signaling, Danvers, USA). For staining of the nucleus, DAPI (1:1000 in 1 mg/mL stock solution in 3% BSA/PBS, Sigma-Aldrich Chemie GmbH, Steinheim, Germany) was used.

### 4.8. In Silico Analysis

To explore potential microRNA-622 targets and pathways, the following databases were used: miRTarBase 2020, miRDB, TargetScan, miRNAMap 2.0 and string [18,19,20,21,52]. For analysis of The Cancer Genome Atlas (TCGA) derived datasets, the Gene Expression Profiling Interactive Analysis (GEPIA) database [53] as well as datasets provided by the human ProteinAtlas database [13,14,15] were applied. The expression data are first log_2_(TPM+1) transformed for differential analysis. Statistical significance was determined by computational log-rank testing and Hazard ratios. The method for differential gene expression analysis was one-way ANOVA.

### 4.9. Statistical Analysis

Results are expressed as mean ± SEM. The unpaired Student’s *t*-test or, if appropriate, the one-way analysis of variance (ANOVA) with Dunnett’s multiple comparison test were used for comparisons between groups (if not depicted otherwise). For analysis of immunohistochemistry scores, the Fisher’s exact test was used. The threshold significance level was *p* < 0.05, with the different levels of significance abbreviated as *: *p* < 0.05, **: *p* < 0.01, ***: *p* < 0.001, ****: *p* < 0.0001 and “ns” for non-significant. Spearman and Pearson correlation coefficients, respectively, were used for correlation analyses. Comparison of survival curves obtained from publicly available databases were conducted in silico using Log Rank-tests and Hazard ratio estimates. Calculations were performed using the GraphPad Prism Software (GraphPad Software, Version 7.0, GraphPad Software, San Diego, CA, USA).

## 5. Conclusions

Our study revealed combined upregulation of the two synergistic and drug resistance associated genes, MAPK14 and ATF2, in HCC and in sorafenib resistant cells. MiR-622 was identified to directly target both genes, resulting in combined de-repression of the MAPK14-ATF2-axis. MiR-622 as well as MAPK14 and ATF2 represent promising potential therapeutic targets in liver cancer.

## Supplementary Materials

The following are available online at https://www.mdpi.com/2072-6694/13/5/1183/s1, Figure S1: Expression patterns of MAPK14 and ATF2 and correlation with tumor stages and survival in HCC, Figure S2: Real-time cell proliferation analysis (RTCA) of sorafenib-resistant HCC cells, Figure S3: The uncropped Western blots, Table S1: Gene expression in paired peri-HCC vs. HCC patient tissues, Table S2: Clinicopathological characteristics and MAPK14 immunoreactivity in human HCC tissues, Table S3: Clinicopathological characteristics and ATF2 immunoreactivity in human HCC tissues.
