# Supplementary material for: Combined De-Repression of Chemoresistance Associated Mitogen-Activated Protein Kinase 14 and Activating Transcription Factor 2 by Loss of microRNA-622 in Hepatocellular Carcinoma
Source: Cancers (Basel). 2021 Mar 9;13(5):1183. doi: 10.3390/cancers13051183 (PMC7967205; doi:10.3390/cancers13051183)
Supplement: Supplementary file 1 [file cancers-13-01183-s001.pdf]

cancers

MDPI

Supplementary Materials: Combined De-Repression of Chemoresistance Associated Mitogen-Activated Protein Kinase 14 and Activating Transcription Factor 2 by Loss of microRNA-622 in Hepatocellular Carcinoma

Valerie Fritz, Lara Malek, Anne Gaza, Laura Wormser, Majken Appel, Andreas E. Kremer, Wolfgang E. Thasler, Jürgen Siebler, Markus F. Neurath, Claus Hellerbrand, Anja K. Bosserhoff and Peter Dietrich

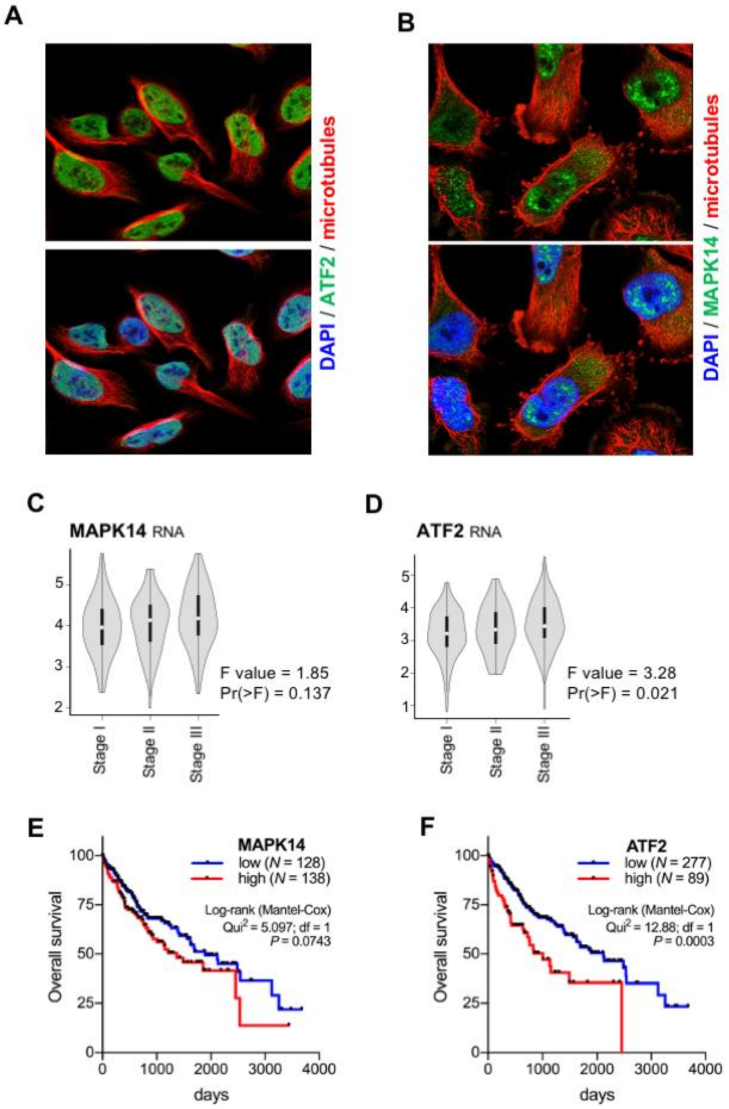

Figure S1. Expression patterns of MAPK14 and ATF2 and correlation with tumor stages and survival in HCC. (**A**,**B**) Immunofluorescence image confirming exclusive nuclear localization of ATF2 (**A**) also in non-HCC cancer cell lines (e.g., the glioblastoma cell line "U-251 MG") (40-fold magnification). (**B**) Immunofluorescence image revealing cellular MAPK14 expression patterns (both cytoplasmatic and nuclear) in non-HCC cancer cell lines (e.g., the glioblastoma cell line "U-251 MG") (40-fold magnification). (**C**,**D**) MAPK14 (**C**) and ATF2 (**D**) RNA expression (log_2(TPM)) different HCC tumor stages (I–III). TCGA-derived data were used applying the Gene Expression Profiling Interactive Analysis (GEPIA) database. (**E**,**F**) "The Cancer Genome Atlas" (TCGA) derived dataset provided by the ProteinAtlas database was used for overall survival analysis comparing high and low MAPK14 (**E**) and ATF2 (**F**) levels, respectively, in HCC patients. Survival analysis was performed computationally applying log-rank testing (Mantel-Cox) (**E**,**F**).

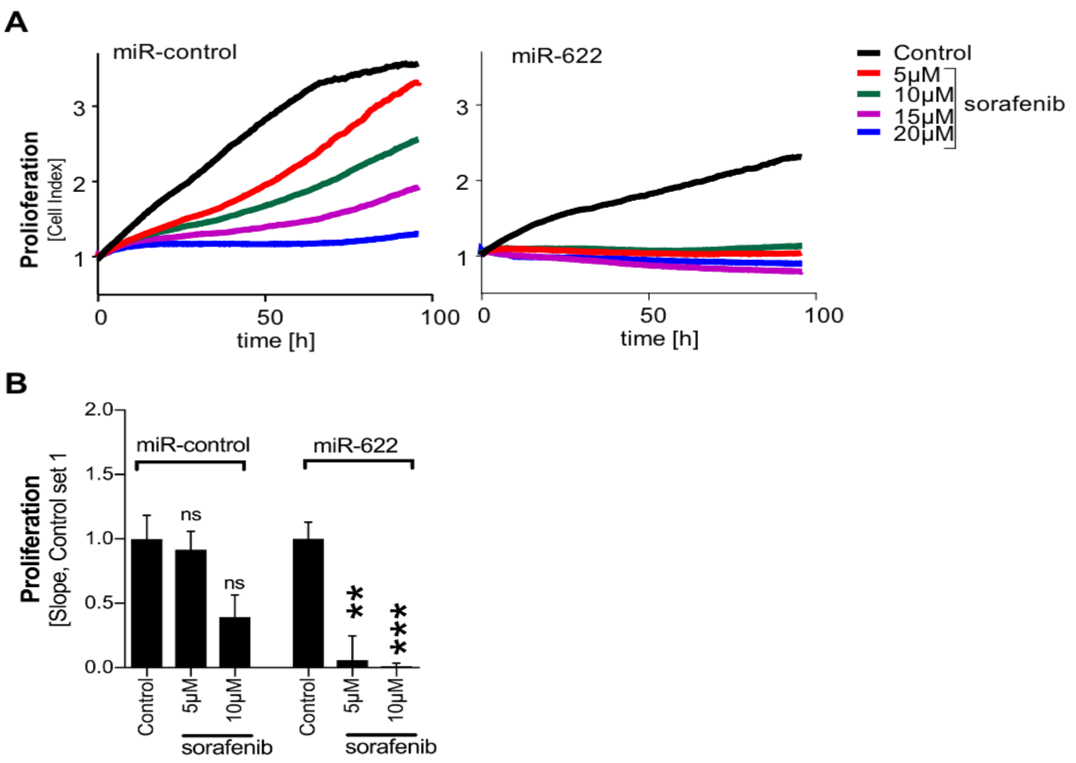

Figure S2. Real-time cell proliferation analysis (RTCA) of sorafenib-resistant HCC cells. (**A**,**B**) Real-time cell proliferation analyis (RTCA) of sorafenib-resistant HCC cells (PLC) treated with different doses of sorafenib (0, 5, 10, 15, 20µM), with (miR-622) or without (miR-control) prior transfection of miR-622. (A) reveals representative proliferation curves. (B) summarizes proliferation (calculated by the mean "slope" of the proliferation curves) (n = 4). Data are presented as the mean ± SEM. Statistical significance was determined by 2-tailed, unpaired t-test (**B**). **P < 0.01 vs Control, ***P < 0.001 vs Control, ns: non-significant vs Control.

Uncropped Western blots

MAPK14 staining

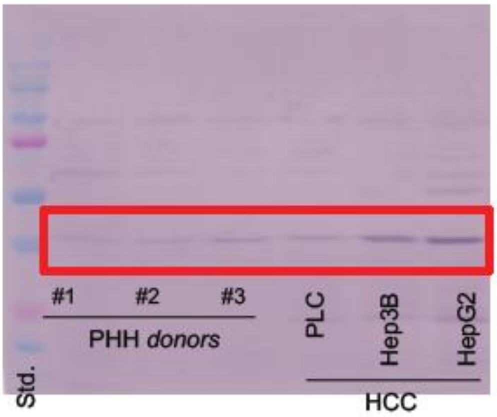

ATF2 staining

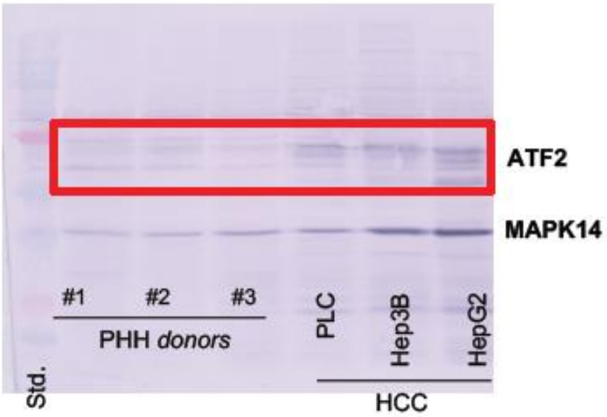

ß-Actin staining

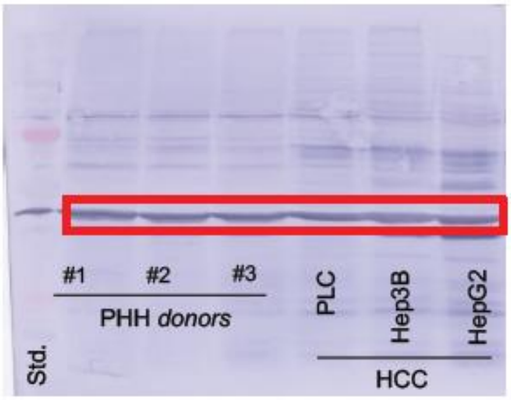

Uncropped Western blot

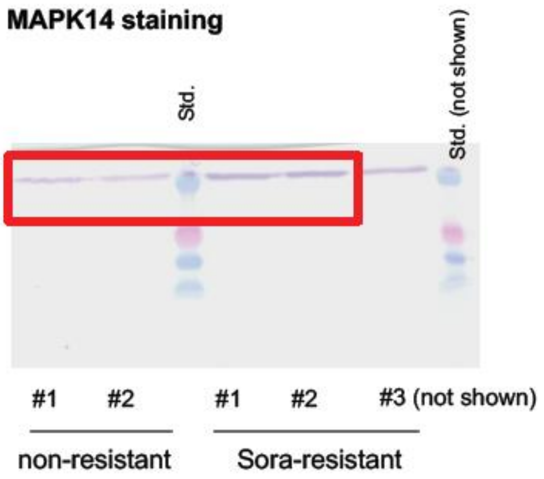

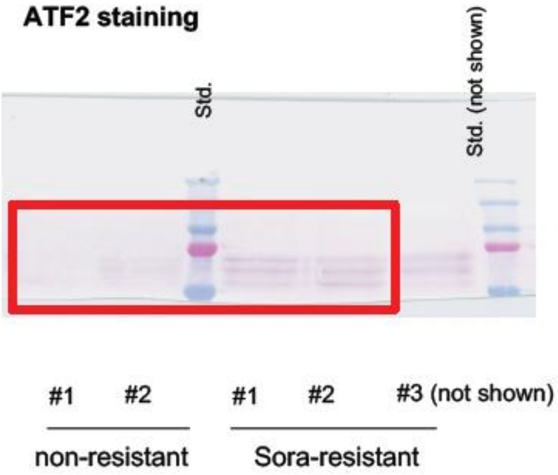

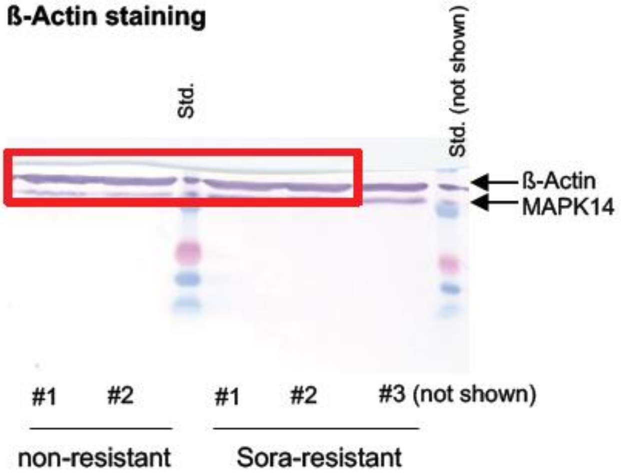

Uncropped Western blot

MAPK14 staining

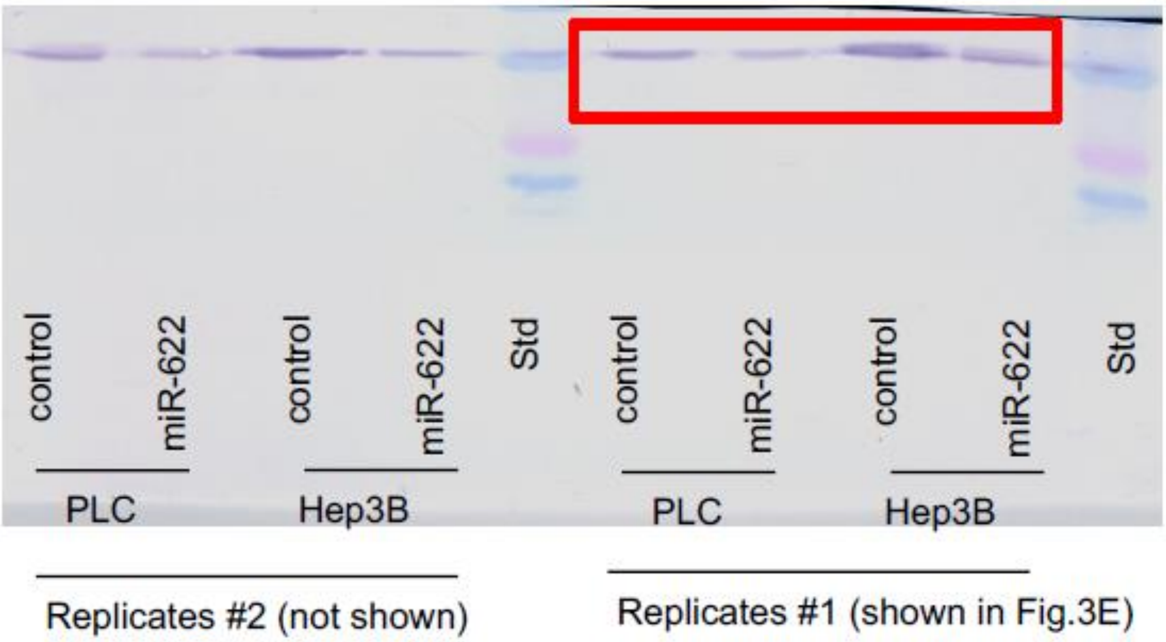

ATF2 staining

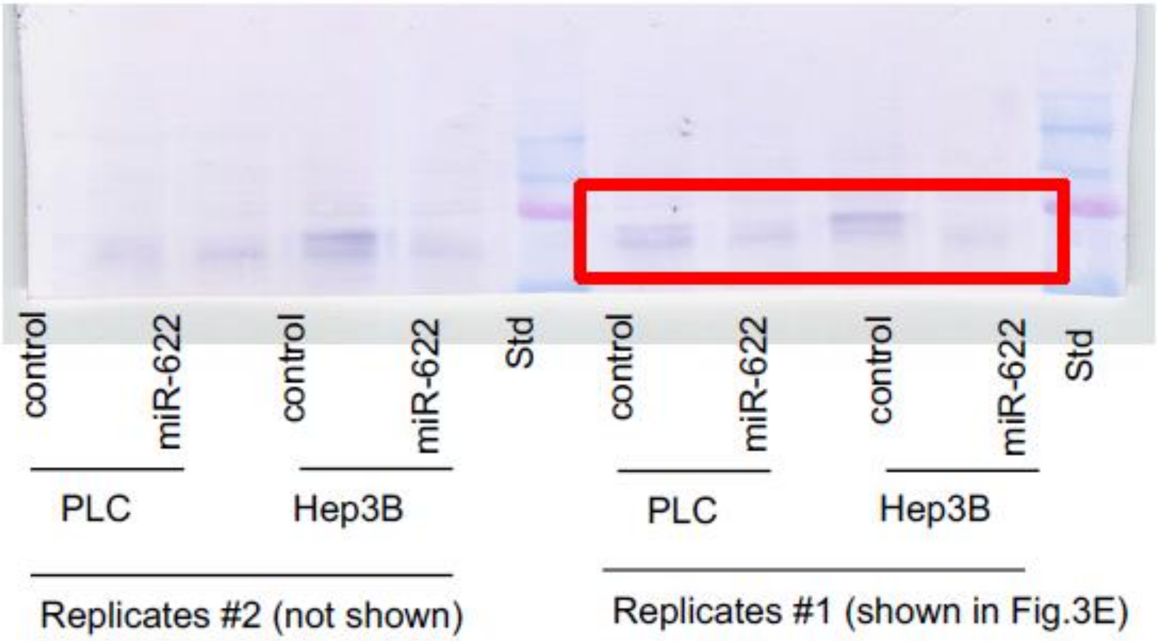

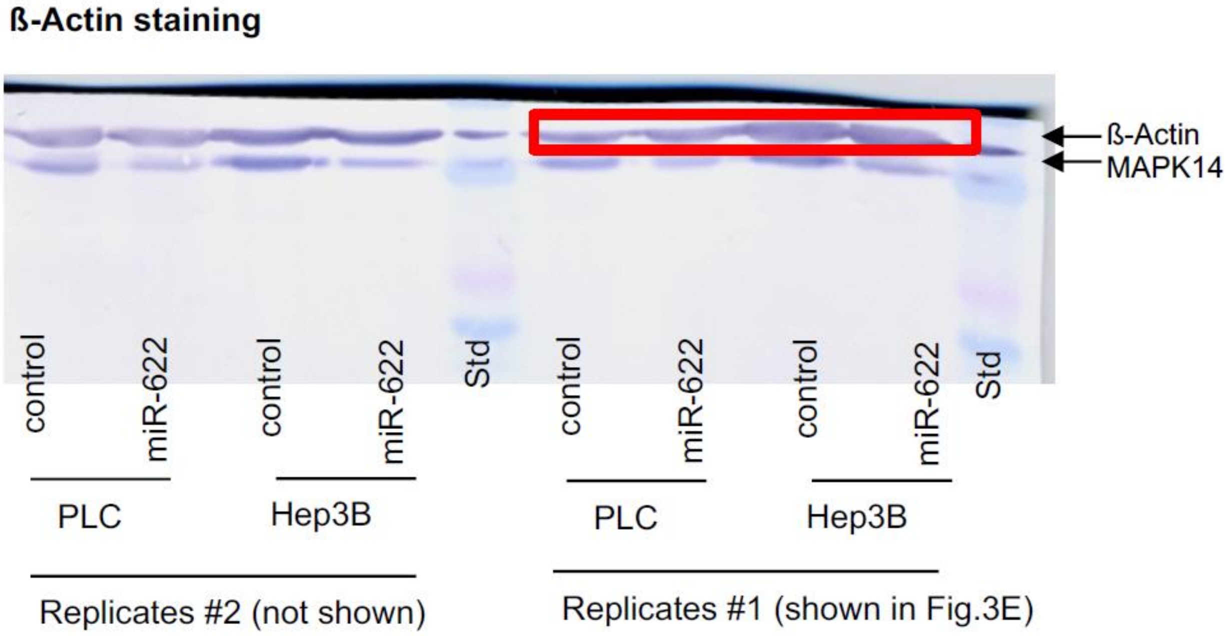

Figure S3. The uncropped Western blots.

Table S1. Gene expression in paired peri-HCC vs. HCC patient tissues.

Gene Name	IR-Score	*n* (%)	peri-HCC	HCC	*P* *	*R* ** *P* **
**MAPK14**	negative/low (0)	43 (46.7)	26	17	**0.006**	0.300
	moderate (1)	34 (36.9)	16	18		**0.004**
	strong (2)	15 (16.3)	2	13		
**ATF2**	negative/low (0)	28 (32.2)	17	11	**0.028**	0.030
	moderate (1)	39 (44.8)	22	17		**0.029**
	strong (2)	20 (23.0)	5	15		

* Fisher's exact test (two-sided); bold face representing *P*-values <0.05. **Spearman correlation (*R*) and according *P*-value (*P*). IR, immunoreactivity. Y5R, NPY, TGFβ and DPP4 staining scores were analyzed qualitatively according to both staining intensity (describing "negative/low" ("0"), "moderate" ("1"), or "strong" ("2")) and percentage of positive cells ("0": <5 %; "1": 5-20%; "2": 20-40%, "3":>40% positive cells).

Table S2. Clinicopathological characteristics and MAPK14 immunoreactivity in human HCC tissues.

Clinico-Pathological Characteristic	Categorisation	MAPK14 IR–Tumor Site					
		n (%)	Negative/Low (0)	Moderate (1)	Strong (3)	*P**	*R* ** *P* **
Age at diagnosis	<60 years<br>≥60 years	12 (25.0)<br>36 (75.0)	6<br>11	4<br>14	2<br>11	0.492	0.185<br>0.224
Gender	Female<br>Male	4 (8.30)<br>44 (91.7)	0<br>17	3<br>15	1<br>12	0.238	−0.136<br>0.398
Fibrosis (Desmet score)	1<br>2<br>3<br>4	7 (14.9)<br>12 (25.5)<br>22 (46.8)<br>6 (12.8)	3<br>2<br>8<br>3	2<br>5<br>9<br>2	2<br>5<br>5<br>1	0.786	−0.153<br>0.303
Aetiology	alcohol<br>non-alcohol (NAFLD, HBV, HCV)	12 (25.0)<br>36 (75.0)	3<br>14	6<br>12	3<br>10	0.645	0.067<br>0.610
**Histological grade**	G1<br>G2<br>G3	18 (36.5)<br>22 (45.8)<br>8 (17.7)	9<br>8<br>0	7<br>8<br>3	2<br>6<br>5	**0.047**	0.403<br>**0.005**
Tumor size	<5 cm<br>≥5 cm	29 (63.0)<br>17 (37.0)	8<br>8	11<br>6	10<br>3	0.325	−0.222<br>0.167
**ATF2** IR (tumor site)	negative/low (0)<br>moderate (1)<br>strong (2)	11 (25.6)<br>17 (39.5)<br>15 (34.9)	8<br>8<br>0	3<br>9<br>5	0<br>0<br>10	**<0.0001**	0.75<br>**<0.0001**

* Fisher's exact test (two-sided); bold face representing *P*-values <0.05. ** Spearman correlation (R) and according *P*-value (P). IR, immunoreactivity; ND, not determined. MAPK14 and ATF2 staining scores were analyzed qualitatively according to both staining intensity (describing "negative/low" ("0"), "moderate" ("1"), or "strong" ("2")) and percentage of positive cells ("0": <5 %; "1": 5-20%; "2": 20-40%, "3":>40% positive cells). The Ki-67 staining score was analyzed qualitatively according to both staining intensity (describing "negative" ("0"), "low" ("1"), "moderate" ("2"), or "strong" ("3")) and percentage of positive cells ("0": <5 %; "1": 5-20%; "2": 20-40%, "3":>40% positive cells). Fibrosis was determined applying the "Desmet" Score System. Ns: non-significant.

Table S3. Clinicopathological characteristics and ATF2 immunoreactivity in human HCC tissues.

**Clinico-Pathological Characteristic**	**Categorisation**	**ATF2 IR–Tumor Site**					
		***n* (%)**	**Negative/Low (0)**	**Moderate (1)**	**Strong (3)**	***P****	***R*** *P*****
Age at diagnosis	<60 years<br>≥60 years	11 (25.6)<br>32 (74.4)	2<br>9	5<br>12	4<br>11	0.907	−0.064<br>0.803
Gender	Female<br>Male	4 (9.30)<br>39 (90.7)	0<br>11	1<br>16	3<br>12	0.347	−0.275<br>0.099
Fibrosis (Desmet score)	1<br>2<br>3<br>4	7 (16.7)<br>9 (21.4)<br>20 (47.6)<br>6 (14.3)	2<br>2<br>5<br>2	3<br>3<br>7<br>3	2<br>4<br>8<br>1	0.966	−0.060<br>0.703
Aetiology	alcohol<br>non-alcohol (NAFLD, HBV, HCV)	11 (25.6)<br>32 (74.4)	2<br>9	4<br>13	5<br>10	0.745	0.137<br>0.455
**Histological grade**	G1<br>G2<br>G3	17 (39.5)<br>18 (41.9)<br>8 (18.6)	6<br>4<br>1	9<br>7<br>1	2<br>7<br>6	**0.046**	0.411<br>**0.007**
Tumor size	<5 cm<br>≥5 cm	24 (58.5)<br>17 (41.5)	5<br>5	8<br>8	11<br>4	0.373	−0.203<br>0.189
**MAPK14** IR (tumor site)	negative/low (0)<br>moderate (1)<br>strong (2)	16 (37.2)<br>17 (39.5)<br>10 (23.3)	8<br>3<br>0	8<br>9<br>0	0<br>5<br>10	**<0.0001**	0.75<br>**<0.0001**

* Fisher's exact test (two-sided); bold face representing *P*-values <0.05. **Spearman correlation (R) and according *P*-value (P). IR, immunoreactivity; ND, not determined. MAPK14 and ATF2 staining scores were analyzed qualitatively according to both staining intensity (describing "negative/low" ("0"), "moderate" ("1"), or "strong" ("2")) and percentage of positive cells ("0": <5 %; "1": 5-20%; "2": 20-40%, "3":>40% positive cells). The Ki-67 staining score was analyzed qualitatively according to both staining intensity (describing "negative" ("0"), "low" ("1"), "moderate" ("2"), or "strong" ("3")) and percentage of positive cells ("0": <5 %; "1": 5-20%; "2": 20-40%, "3":>40% positive cells). Fibrosis was determined applying the "Desmet" Score System. Ns: non-significant.
